# Competition and Habitat Quality Influence Age and Sex Distribution in Wintering Rusty Blackbirds

**DOI:** 10.1371/journal.pone.0123775

**Published:** 2015-05-06

**Authors:** Claudia Mettke-Hofmann, Paul B. Hamel, Gerhard Hofmann, Theodore J. Zenzal Jr., Anne Pellegrini, Jennifer Malpass, Megan Garfinkel, Nathan Schiff, Russell Greenberg

**Affiliations:** 1 School of Natural Sciences and Psychology, Liverpool John Moores University, Liverpool, United Kingdom; 2 US Forest Service, Stoneville, MS, United States of America; 3 Tamar Grove, Moreton, United Kingdom; 4 Department of Biological Sciences, University of Southern Mississippi, Hattiesburg, United States of America; 5 SWCA Environmental Consultants, Flagstaff, AZ, United States of America; 6 School of Environment and Natural Resources, Ohio State University, Columbus OH, United States of America; 7 Department of Wildlife, Humboldt State University, Arcata CA, United States of America; 8 Smithsonian Migratory Bird Center, National Zoological Park, Washington, D. C., United States of America; Universidad de Granada, SPAIN

## Abstract

Bird habitat quality is often inferred from species abundance measures during the breeding and non-breeding season and used for conservation management decisions. However, during the non-breeding season age and sex classes often occupy different habitats which suggest a need for more habitat-specific data. Rusty Blackbird (*Euphagus carolinus*) is a forested wetland specialist wintering in bottomland hardwood forests in the south-eastern U. S. and belongs to the most steeply declining songbirds in the U.S. Little information is available to support priority birds such as the Rusty Blackbird wintering in this threatened habitat. We assessed age and sex distribution and body condition of Rusty Blackbirds among the three major habitats used by this species in the Lower Mississippi Alluvial Valley and also measured food availability. Overall, pecan groves had the highest biomass mainly driven by the amount of nuts. Invertebrate biomass was highest in forests but contributed only a small percentage to overall biomass. Age and sex classes were unevenly distributed among habitats with adult males primarily occupying pecan groves containing the highest nut biomass, females being found in forests which had the lowest nut biomass and young males primarily staying in forest fragments along creeks which had intermediate nut biomass. Males were in better body condition than females and were in slightly better condition in pecan groves. The results suggest that adult males occupy the highest quality habitat and may competitively exclude the other age and sex classes.

## Introduction

Habitat requirements differ for many migratory bird species between the breeding and nonbreeding season [1, 2] and a species is often found in several habitats during the nonbreeding season (e.g. [3, 4, 5]). Interestingly, age and sex classes often occupy different habitats [6, 7, 8] which has been linked to social dominance-mediated competitive exclusion related to differences in habitat quality [2, 9, 10]. However, a few studies have also shown sex-specific habitat preferences (e.g. [11]). Segregation into habitats of different quality not only affects individual fitness through carry-over effects of winter conditions into the next breeding season but also demographic development when one sex (usually the female) is pushed into lower quality habitats [12, 13].

An increasing number of bird species, particularly migrants, show negative population trends [14, 15]. To counter this, conservation efforts often target habitats as their availability and quality critically affects population development throughout the annual cycle. Habitat suitability is measured in terms of species richness and abundance [16, 17], but to account for age and sex segregation more habitat-specific data are necessary [2].

Birds of wooded wetlands are particularly prone to decline in the face of anthropogenic change [18] as loss and degradation of this habitat type are affected by logging, agricultural development, changes in hydrology, and contamination [19]. An example is the Rusty Blackbird (*Euphagus carolinus*), who breeds in wetlands within the boreal forest of Canada and the U.S. and winters primarily in bottomland hardwood forests in the south-eastern United States [20]. This species exhibits the steepest decline of any North American songbird with an estimated decline of -12.5% / yr from Breeding Bird Surveys (BBS, [21]) and -4.5% / yr from Christmas Bird Counts (CBC, [22]) accumulating to a 85–95% loss of the population over the last 40 years. Part of this decline coincides with considerable loss in bottomland hardwood forests since the 1940s [23, 24] due to logging and conversion to agricultural land. Other contributing factors include habitat loss and changes on the breeding grounds [25] as well as climate change [26].During winter, Rusty Blackbirds primarily occur in swamps, wet woodlands, pond edges, and hardwood forests, where they feed on acorns and pecan nuts on the ground but also on invertebrate prey hidden in the leaf litter, in pools, and under floating plants [20]. However, more detailed habitat use and requirements are unknown which makes it difficult to assess the role of habitat change on past and ongoing population declines. It also limits our ability to recommend improvements to reforestation efforts and water management strategies to benefit Rusty Blackbird habitat. This lack of specific knowledge is particularly evident when considering that bottomland hardwoods have not only decreased in size but that the remaining forest is characterized by fragmentation, changed hydrology and reforestation with selected tree species [19].

The Lower Mississippi Alluvial Valley is the core wintering area for Rusty Blackbirds, supporting on average more than double the abundance of Rusty Blackbirds than any other of the 35 Bird Conservation Regions where the species was detected [22]. A pilot study conducted in the vicinity of Greenville, Mississippi, during the winter 2003–2004 indicated that Rusty Blackbirds consistently use three habitats—bottomland hardwood forests, forest fragments along creeks (i.e. trees bordering creeks), and partially harvested pecan (*Carya illinoinensis*) groves. While Rusty Blackbirds are not territorial during winter, competition about food resources is very likely to occur in groups [27]. Differences in habitat quality may lead to segregation between age and sex classes [28], particularly as the species shows sexual size dimorphism [20]. The present study, therefore, aimed to a) investigate distribution of age and sex classes and b) assess body condition of Rusty Blackbirds as well as c) measure food availability in the three habitat types regularly used by this species to gain a detailed knowledge about habitat quality and use. Knowledge about habitat quality may provide important information for other species of concern including wintering ducks [29], Common Grackles (*Quiscalus quiscula*) for which declines have been recently reported [30] and American Robins (*Turdus migratorius*) [31]. Therefore, this study has a wider applicability with respect to bottomland hardwoods as winter habitat for birds.

## Material and Methods

### Study site and data collection

**Fig 1:**
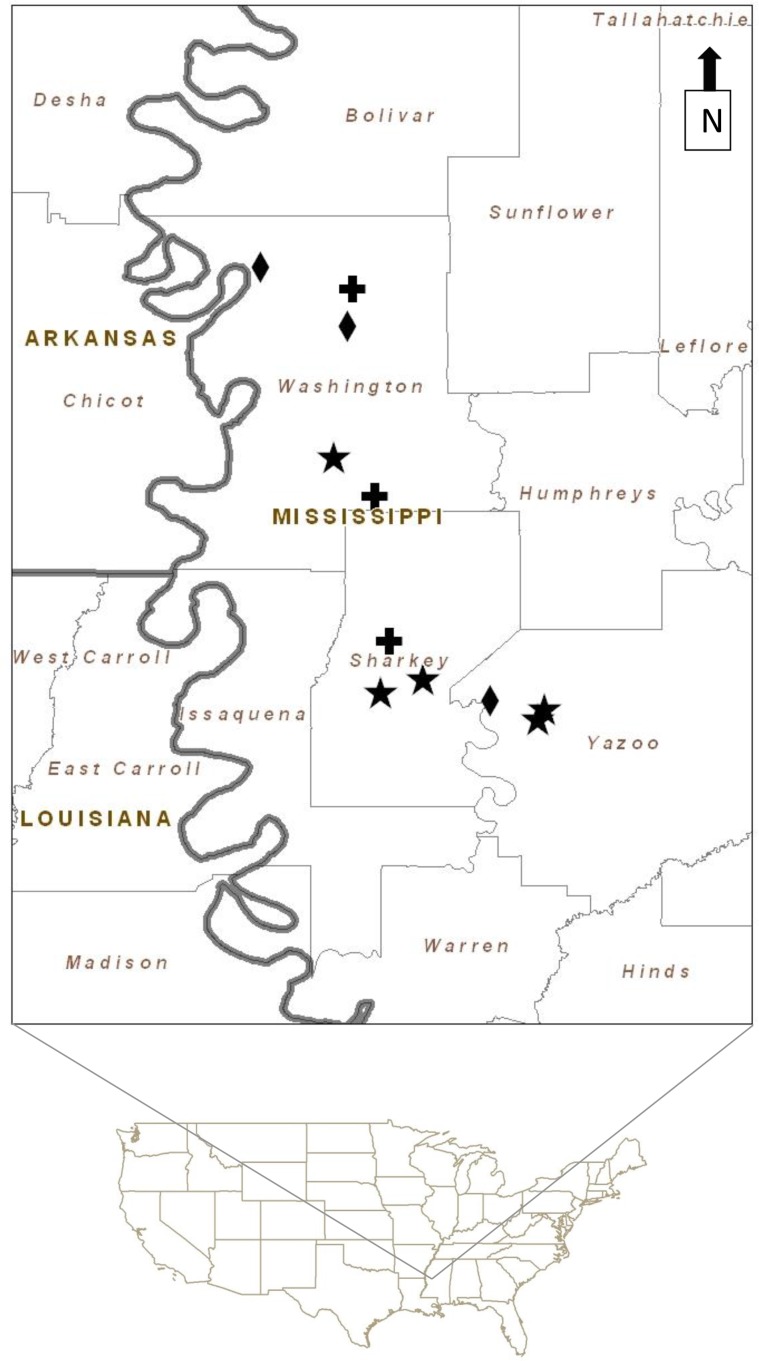
Sampling sites in the Lower Mississippi Alluvial Valley. The location of the study site in the United States is shown (lower part) with sampling sites indicated according to the habitat (upper part). Diamond: Pecan grove locations, cross: creek locations, stars: forest locations; the gray line represents the Mississippi River.

The study was conducted in the Lower Mississippi Alluvial Valley near Greenville, Mississippi (33°27.290’N, 91°02.093’W), in the core winter area of Rusty Blackbird. Birds were captured in an area of 80 km in north-south direction and 40 km in east-west direction along the Mississippi River covering approximately 3200 km^2^ (Fig 1) during three consecutive winters from early December to the end of March 2005–2008. Further capture data were available from Tensas River NWR (32.2500° N, 91.3667° W; about 100 km south of Greenville; provided by Dan Twedt) and a bottomland hardwood patch along a pond in Washington County, Arkansas (36° 8'44.20"N, 94°10'35.34"W; 500 km northwest of Greenville; provided by J. D. Luscier) during the same period. We captured Rusty Blackbirds in the only habitats consistently used by the species (as found during the pilot study, 2003–2004): bottomland hardwood forests, partially harvested pecan groves, and forest fragments along creeks. Capture locations were at least 5 km apart.

Overall, six capture locations were placed in forests (Leroy Percy State Park, Panther Swamp National Wildlife Refuge (NWR) (*n* = 2), Delta National Forest (*n* = 2), Tensas River NWR), four in forest fragments along creeks (including the forest fragment in Arkansas) and three in pecan groves (Fig 1). All forest locations were in extended bottomland hardwood forest with leaf litter cover, varying amounts of understory and variable water cover on the ground depending on weather. Forest fragments along creeks had a mixture of dense and open understory along the edge of the creek with leaf litter cover on the ground and floating plants on the water but were otherwise bordered by roads, fields and houses. Creeks had variable water levels which fluctuated with rainfall. Pecan groves were very open with basically no understory and often had one or two oak trees included. These groves were only partially harvested providing pecan nuts throughout the winter. This habitat type was the driest only having puddles after rain.One creek location was used for capture every winter and one grove and one forest location in the winter of 2005/ 2006 and 2006/ 2007. Otherwise capture locations differed between years but always included sites from all three habitats. Capture sites for Rusty Blackbirds were localized during reconnaissance surveys. Usually, several candidate locations were identified for each habitat. Those fulfilling the following criteria were chosen: capture sites were at least five kilometres away from any other capture site used during the study; were accessible by car (as baiting—see below—took place in the dark and captured birds were measured in the car to avoid disturbance to other birds); and were frequented by Rusty Blackbirds regularly. Rusty Blackbirds are difficult to capture, so to improve our chances we baited most areas with custom-made egg food (crushed boiled eggs mixed with cracked corn and corn meal) scattered on the ground. At two sites (one in a forest (Tensas River NWR, *n* = 20 birds), the other in a forest fragment along a creek, *n* = 11 birds) birds were captured without food. No differences in condition index and body mass of birds were found between these unbaited sites and baited sites (forest *n* = 20 birds, creek *n* = 8 birds) of the same habitat type sampled in the same year (t-tests, all *P*>0.15). Furthermore, visual inspection of age/ sex composition did not reveal differences between baited and unbaited sites. Birds were captured with mist nets (38mm mesh) starting at dawn and continuing for approximately 3 hours. At each site we made an average of 5 capture attempts (± 3 SE) separated by an average of 8 days (± 6 SE) without capture activity. A total of 208 Rusty Blackbirds were captured during the study. While capture effort differed between years (mean net hours 157h ± SE 74h) depending on number of people available and difficulty in catching birds, at least one site in each habitat was sampled in each year. Capture effort (net hours) did not differ between habitats (Kruskal-Wallis: z = 0.7895, df = 2, *P* = 0.6739).

We determined age as younger (young), or older (adults) than one year and sex following Pyle [32] and took several morphological measurements including unflattened wing chord, tarsus length, pectoral muscle breadth [33], fat score (mean of fat score (range 0–8) of interclavicula and abdomen, [34]) and body mass. Birds were banded with a standard USGS metal band and an individual color band combination. Due to other commitments little effort was put into resightings but all resightings (20 different birds out of 122 banded birds in 2005/2006 and 2006/2007) were near the site of banding and these birds persisted there over the course of the winter. Additionally, telemetry data (*n* = 49 birds) collected over one-month periods between 2006 and 2010 indicate that the birds were site faithful at least over a month. No Rusty Blackbirds were recaptured.

Rusty Blackbirds are found in habitats characterized by Willow Oak (*Quercus phellos*), Water Oak (*Q*. *nigra*), Overcup Oak (*Q*. *lyrata*), Nuttall Oak (*Q*. *nuttalli*) and Pecan (C. Mettke-Hofmann unpubl. data). Acorns in general [20], and small acorns in particular, are a preferred food by Rusty Blackbirds together with pecan nuts (C. Mettke-Hofmann personal observation). Additionally, this species consumes a higher proportion of invertebrates in winter than other blackbird species [20]. Invertebrates are particularly searched along water edges of ponds and streams often wading in shallow water turning leaves. Dry leaves on land are also flipped to find food [20]. In the winters 2008/ 2009 and 2009/ 2010 invertebrate and acorn/ pecan nut abundances were sampled in the different habitats on a bi-weekly basis from mid-December to mid-March. Fourteen different sites were sampled in 2008/ 2009 (5 in pecan groves, 5 in forest fragments along creeks and 4 in forests) and 20 in 2009/ 2010 (7, 8, 5 sites, respectively). The chosen sites represented core foraging areas of Rusty Blackbird determined from telemetry data (C. Mettke-Hofmann unpubl. data). Each site was sampled six to nine (average 7.5) times in 2008/ 2009 and nine to ten times (average 9.9) in 2009/ 2010. Within sites sampling locations were selected at random. Invertebrate abundance was sampled by collecting all leaves on the ground in an area of 25 x 25 cm in a single swipe with a shovel to catch all ground-dwelling invertebrates. The shovel was placed below the leaf litter but just above the soil. Samples were stored in a sealed plastic bag. Invertebrate samples from wet (if available) and dry areas were taken. Invertebrates were then extracted by turning each leaf and all specimens were collected and preserved in alcohol for later identification to the lowest taxonomic level (often family or genus). Abundances of all invertebrate taxa were recorded. A representative sample of all invertebrates was dried for 24 hours at 70°C in a Fisher Isotemp oven and weighed. Abundance of acorns/ pecans was assessed by counting all nuts on the ground in an area of 50 x 50 cm in all three habitats. Sampling areas within a site were always placed at random by selecting a different area within a site for each sampling and then sampling beneath the canopy of the first oak or pecan tree encountered. If both oak and pecan trees were available at the same site, two samples were taken. Nut samples of willow oak, water oak, wild and cultivated pecan were dried for 48 hours at 50 degrees in a Fisher Isotemp oven and weighted (kernel only without shell).

### Analyses

We used hierarchical log-linear analyses to compare age and sex composition among the three main habitats and years (*n* = 207 birds as one bird could not be aged). Log-linear models allow examining relationships between categorical data by analysing multi-way contingency tables without distinguishing between dependent and independent variables [35], i.e., age and sex can be analysed in the same model. Hierarchical log-linear models consider nestedness of data (e.g. sites within habitats, habitats within years etc.) and are a special case of general mixed linear models [36]. This type of analysis first generates a saturated model with all variables included and then a stepwise procedure in model selection is used [35]. Here we used backward elimination to remove variables. The variable with the largest observed significance level for the change in chi-square is removed as long as it does not change the chi-square value significantly [37]. Due to the hierarchical structure of analysis, higher-order terms include all lower-order terms [35].

A body condition index was calculated as the quotient of body mass to wing length [38]. Morphological data were available for 203 birds. Additionally, body mass to tarsus length (*n* = 186) and pectoral muscle breadth to wing length (*n* = 125) ratios were calculated. Body mass/wing length was correlated with both other measures of condition (Pearson’s correlation body mass/tarsus *r* ^2^ = 0.78, *P*<0.001; body mass/pectoral muscle *r* ^2^ = 0.23, *P* = 0.009). Condition index did not correlate with time of capture (Pearsons correlation *r* = 0.12, *P* = 0.10). Further analyses only used the condition index based on body mass to wing length as this index was available from nearly all birds. We used ANOVA to test whether body condition index differed between age and sex classes, habitats and years with capture location as a random factor. LSD posthoc tests were performed to test for differences between habitats. As there was a highly significant difference in body condition index between sexes we a) ran the same analysis for both sexes separately and b) further tested whether this difference was possibly caused by different morphometrics between sexes [39] or indeed due to differences in body condition. For the latter, we included two independent measures of body condition: fat score (*n* = 186) and pectoral muscle breadth (*n* = 125; Pearson correlation *r* = -0.019, *P* = 0.83), into an ANOVA to test whether these factors explained any variation in body condition index.

To analyse food availability data, we calculated mean abundances for invertebrates and nuts (pecan or acorn) across the winter at each site to get an overall estimate of available biomass. For invertebrate samples the mean of wet and dry sites was taken with wet sites set to zero when no wet patches were present as these represent an important foraging micro-habitat [20]. We calculated approximate dry invertebrate biomass of 10 taxa (ant, beetle, isopod, larva, leech, mussel, shrimp, snail, spider and worm—which were all known to be eaten by Rusty Blackbirds ([20], C. Mettke-Hofmann personal observation) by multiplying their mean dry biomass with their respective abundances. To scale dry invertebrate biomass to a 50 x 50 cm plot comparable to that of the nuts, all values were multiplied by four as a final step. For nut samples most sites had either pecan or acorns but in cases where samples from both trees were taken we used the higher value for analysis. Nut biomass was calculated for acorn (willow and water oak), wild and cultivated pecan by multiplying their mean dry biomass with their respective abundances. We analysed years separately because abundances of nuts differed significantly between 2008/ 2009 and 2009/ 2010 (U-test: *P* = 0.005) and differences in invertebrate abundances between years approached significance (*P* = 0.093). We used ANOVAs to compare invertebrate and nut biomass between habitats and used LSD post hoc tests. We also conducted ANOVAs for the wet and dry samples separately to investigate their contribution to the results. All analyses were completed in IBM SPSS Statistics 20.0 (IBM Corporation, Armonk, NY, U.S.A). Original data are provided as Supporting Information for capture and body condition analyses (S1 Table) and food availability (S2 Table).

### Permits

Work permits were given to Claudia Mettke-Hofmann for Yazoo National Wildlife Refuge (Special Use Permit No. 04005 US Department of the Interior, Fish and Wildlife Service), Leroy Percy State Park (verbal agreement by Park Authority (Mississippi Department of Wildlife, Fisheries and Parks), Delta Research & Extension Center land (agreement given via email by Delta Research & Extension Center) and Delta National Forest (DNF) and public land outside of the DNF in the Mississippi Delta (Administrative Scientific Collection Permit (letter) issued by Mississippi Museum of Natural Sciences). Furthermore, all work conducted on private land was permitted by the farmers involved. Birds were captured and banded under the Federal Bird Banding permit (US Department of the Interior) No. 09613 and the Federal Fish and Wildlife permit MB116210-0, both issued to Paul B. Hamel.

## Results

### Age and sex composition in relation to habitat and year

**Fig 2:**
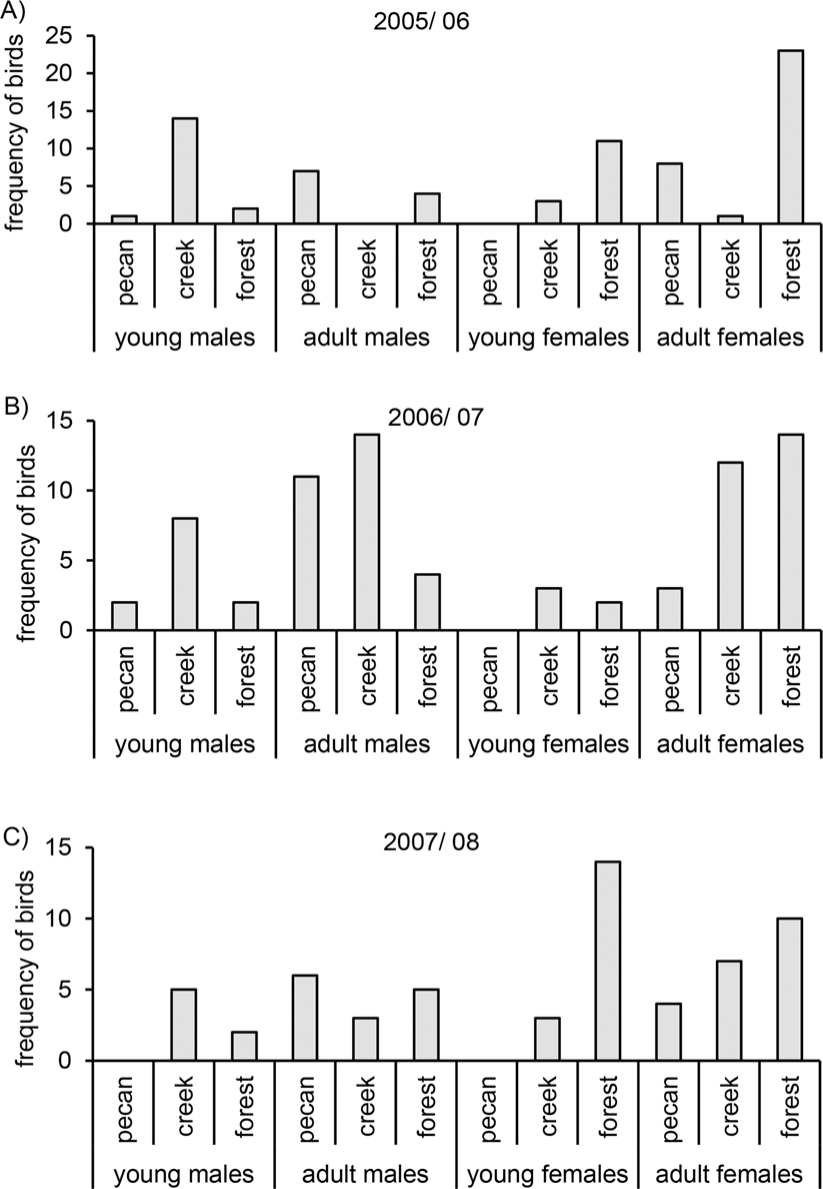
Age and sex composition in different habitats. Frequency of age and sex classes captured in pecan groves, forest fragments along creeks and forests in three consecutive winters. Frequencies of age and sex classes were summed up for sites of the same habitat type within a given year.

We caught substantially more adults (*n* = 136) than young (*n* = 71) and a majority of captures were females (*n* = 118; males *n* = 89), so that the largest single age/sex class was adult females (n = 82), followed by adult males (*n* = 54) and similar numbers of young of both sexes (*n* = 36 females, 35 males, Table 1: sex x age interaction). In the final model, the highest order terms retained were a significant interaction between age x habitat x year and sex x habitat (Table 1) showing that sex and age classes occurred with different frequencies in the three habitats. Young males were mainly found in forest fragments along creeks and adult males in pecan groves, both particularly in 2005/2006 and 2007/2008 (Fig 2). Both young and adult females were mainly found in forests throughout the study period. However, in 2005/2006 and 2007/2008 adult females were also found in pecan groves and forest fragments along creeks (Fig 2). Furthermore, abundance of age classes varied between years (age x year interaction) with relatively fewer young in 2006/ 2007 than in the other years (Table 1, Fig 2). The significant interaction between habitat x year indicates that although we captured birds in all three habitat types each year, habitats were not sampled with equal intensity (i.e. number of capture sites).

**Table 1:** Results of the hierarchical log-linear analysis for age and sex composition in different habitats and years.

Maximum likelihood chi-square			
K-way and higher order effects			
	df	chi^2^	*P*
1^st^ order effects	35	171.054	< 0.001
2^nd^ order effects	29	124.201	< 0.001
3^rd^ order effects	16	32.226	0.009
4^th^ order effects	4	1.707	0.789
Goodness of fit (final model)	15	18.209	0.252
**Partial association**			
	df	chi^2^	P
sex x age x habitat	2	3.277	0.194
sex x age x year	2	0.698	0.705
sex x habitat x year	4	2.399	0.663
age x habitat x year	4	13.377	0.010
sex x age	1	4.219	0.040
sex x habitat	2	34.058	< 0.001
age x habitat	2	32.673	< 0.001
sex x year	2	2.662	0.264
age x year	2	9.775	0.008
habitat x year	4	11.143	0.025
sex	1	4.076	0.043
age	1	19.481	< 0.001
habitat	4	21.063	< 0.001
year	2	2.233	0.327

Initially, a saturated model is generated with all variables included, followed by backward elimination of variables. Only variables that do not change the chi-square value significantly are removed. K-way and higher order effects are presented followed by the goodness of fit for the final model. Partial associations which are adjusted for all other effects in the model are shown for all factors and their interactions.

### Body condition index

**Fig 3:**
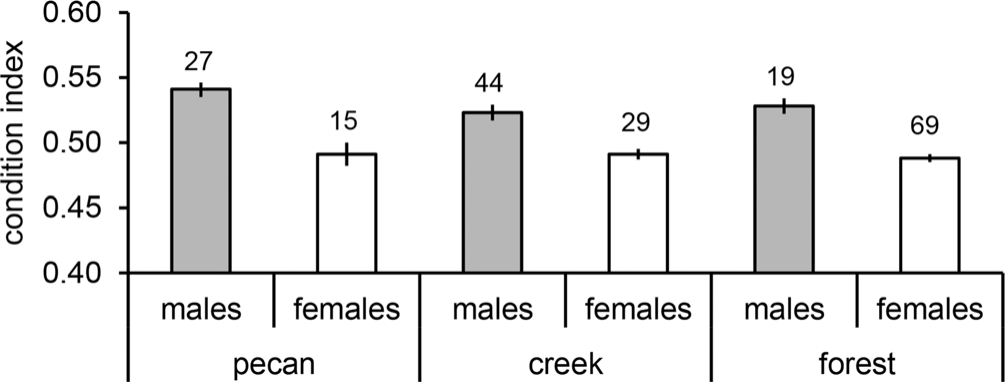
Body condition of males and females. Mean body condition (± SE) for males and females in pecan groves, forest fragments along creeks and forest are shown. Body condition represents the quotient of body mass to wing length. Numbers on top of bars indicate sample sizes. Gray bars: males; white bars: females.

Body condition showed a significant relationship to sex (*F* = 125.002, df = 1, *P*<0.001), habitat (*F* = 3.941, df = 2, *P* = 0.02) and the interaction age x habitat (*F* = 3.047, df = 3, *P* = 0.03) explaining 46.8% of the variance (ANOVA *F*_6,196_ = 30.632, *P*<0.001). Males had a much better body condition than females in all three habitats (Fig 3). Furthermore, body condition was better for birds in pecan groves (*P*<0.001) and along creeks (*P*<0.001) as compared to forests. Finally, young birds in pecan groves had a better body condition (0.57 ± 0.01, *n* = 3) than adult birds (0.52 ± 0.01, *n* = 24) in this habitat, whereas body condition did not differ between age classes in the other habitats. However, only three young birds were captured in pecan groves. No other variables or interaction terms had a significant influence on body condition. Analysing males alone produced the same result (ANOVA *F*_5,84_ = 2.645, *P* = 0.03; habitat *F* = 3.363, df = 2, *P*<0.001; age *F* = 4.399, df = 1, *P* = 0.04; habitat x age *F* = 4.796, df = 2, *P* = 0.01) with age showing a difference in condition of just 0.002 (*n* = 36 and *n* = 54, respectively) between young and old birds which can be considered as biologically negligible. Posthoc tests for the interaction term showed that males in pecan groves tended to have a better body condition than males in forests (*P* = 0.08), whereas no differences in male body condition were found between the other two habitats (all *P*>0.14; Fig 3). In females, only age had an effect with adult females having a better body condition (mean 0.493 ± 0.003SE, *n* = 78) than young females (0.482 ± 0.004, *n* = 35; ANOVA *F*_1,111_ = 4.104, *P* = 0.045).

A further ANOVA was run with sex as factor, fat score and pectoral muscle breadth as covariates (continuous variables)and body condition as the dependent variable to test for a relationship among these variables. In addition to the relationship between body condition and sex, fat score also had a positive relationship with body condition (ANOVA *F*_5,119_ = 41.005, *P*<0.001; sex: *F* = 41.274, df = 1, *P*<0.001; fat score *F* = 20.564, df = 1, *P*<0.001). Furthermore, males had more fat than females (1.5 vs. 1.1, *n* = 90 and *n* = 96, respectively; sex x fat score; *F* = 5.182, df = 1, *P* = 0.03) indicating that differences in condition index were not solely due to differences in morphometrics between sexes. Finally, body condition index in males was influenced by pectoral muscle breadth and fat score, whereas in females body condition index was primarily related to fat score (sex x fat score x pectoral muscle score; *F* = 7.197, df = 2, *P* = 0.001). Overall, 61.7% of the variance in body condition was explained.

### Food availability

**Fig 4:**
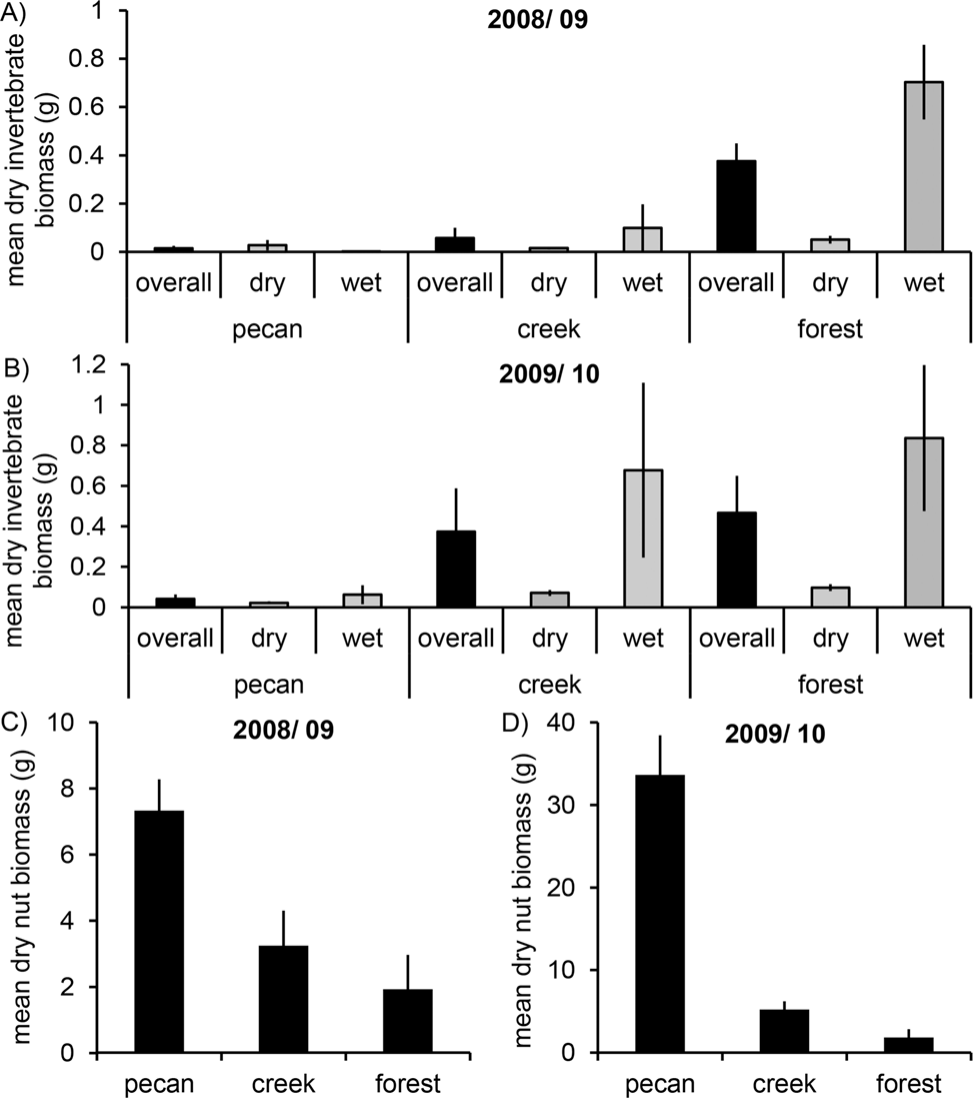
Food availability in different habitats. Mean (± SE) dry invertebrate (A, B) and dry nut biomass (C, D) are shown for two winters (2008/ 2009 and 2009/ 2010) in pecan groves (*n* = 5 and *n* = 7), forest fragments along creeks (*n* = 5 and *n* = 8) and forests (*n* = 4 and *n* = 5). For invertebrates, overall mean biomass (mean of wet and dry sites; black bars) and mean biomass for dry and wet sites (gray bars), respectively, are shown. Nut biomass includes pecan and acorn nuts. Mind the different scales in plot C and D.

In winter 2008/ 2009 mean invertebrate biomass (wet and dry samples together) differed between habitats (ANOVA *F*_2,11_ = 15.621, *P* = 0.001) explaining 69.2% of the variance in invertebrate biomass. Pecan groves (*P*<0.001) and creeks (*P* = 0.001) had significantly less invertebrate biomass than forests (Fig 4A). This was primarily driven by differences in wet samples (*F*_2,11_ = 14.278, *P* = 0.001) with forest having a much higher invertebrate biomass than the two other habitats (pecan—creek *P* = 0.478, pecan—forest *P*<0.001, creek—forest *P* = 0.001). Dry invertebrate samples did not differ in biomass between habitats (*F*_2,11_ = 1.222, *P* = 0.332). In contrast, overall invertebrate biomass did not differ between habitats in winter 2009/ 2010 (*F*_2,17_ = 1.679, *P* = 0.22; Fig 4B). Again this was driven by the wet samples (*F*_2,11_ = 1.393, *P* = 0.275), whereas biomass of dry samples differed significantly between habitats (*F*_2,11_ = 7.729, *P* = 0.004) with fewer invertebrates in pecan groves than the two other habitats (pecan—creek *P* = 0.013, pecan—forest *P* = 0.002, creek—forest *P* = 0.200). The overriding effect of the wet samples is not surprising given their high contribution to overall biomass (Fig 4A and 4B). Nut biomass differed significantly between habitats in both winters (2008/ 2009: *F*_2,11_ = 7.591, *P* = 0.008, *r* = 0.50; 2009/ 2010: *F*_2,17_ = 33.113, *P*<0.001, *r* = 0.77) with pecan groves having consistently higher values than forests (2008/ 2009 *P* = 0.004 and 2009/ 2010 *P*<0.001) and creeks (*P* = 0.01 and *P*<0.001, respectively) with creeks in between the two other habitats (Fig 4C and 4D).

## Discussion

We studied age and sex distribution and body condition of Rusty Blackbirds wintering in three different habitats in Mississippi and assessed habitat quality in terms of food availability. Age and sex classes of wintering Rusty Blackbirds segregated into different habitats with adult males being primarily found in pecan groves which had the highest nut biomass of all habitats, whereas females were mainly found in forests with the lowest amount of nut biomass. Young males occurred primarily in forest fragments along creeks which had intermediate amounts of nut biomass. Males were in better body condition and had larger fat reserves than females. Differences in body condition between habitats were small and mainly driven by better body condition of males in pecan groves. Although distribution of birds and body condition were assessed in different years than food availability, the latter ranked habitats consistently in both years. This indicates that while there is variation in overall food availability between years, habitat differences may persist across years. We therefore, assume that the differences in food availability found between habitats in 2008/ 2009 and 2009/ 2010 are also applicable to the winters 2005–2008 when we sampled Rusty Blackbirds.

### Habitat use of age and sex classes in relation to food availability

In recent years, age and/ or sex segregation into different habitats during the nonbreeding season has been shown for several songbird species [3, 12, 40] and has often been related to differences in food availability with males or adults occupying better habitats [2, 9, 13]. The results of the current study indicate that similar mechanisms may drive distribution of age/ sex classes in the Rusty Blackbird, a species that is not territorial but forms flocks in winter. Although biomass of invertebrates and nuts showed opposite trends in the three habitats overall food biomass differed among habitats. Pecan groves had a higher biomass (driven by nuts) than the other two habitats with equal contribution of nuts and invertebrates along creeks and a higher biomass of invertebrates than nuts in forests (Fig 4). Rusty Blackbird often forages on nuts in the morning and switches to invertebrates later in the day (own obs. C. Mettke-Hofmann). Nuts may provide an important and relatively stable resource (over days and weeks) to refuel energy reserves after the night and during cold spells. In contrast, invertebrate availability may be more variable over the winter as invertebrate abundance depends on temperature [41, 42] and fluctuates with water cover on the ground [43, 44]. The high biomass of nuts in pecan groves would make this a preferable habitat. This aspect should be investigated further.

Given that males are larger than females and adult individuals are likely more competitive than younger ones the most competitive age/ sex class, adult males, occupied the habitat with the highest nut biomass, whereas females, particularly young ones, the least competitive class, were primarily found in forests which had the lowest amount of nut biomass. This may indicate that adult males displaced females and young males from the habitat with the most abundant and stable food source. Alternatively or in addition to intraspecific competition, competition with the larger Common Grackle may exclude females and young Rusty Blackbirds from pecan groves which are particularly open with little understory so that a bird with food is easily detected. Indeed, chases to get food were frequently observed among Rusty Blackbirds and between Common Grackles and Rusty Blackbirds in pecan groves. Forest fragments along creeks are in part open but overall provide much more cover than pecan groves. The relatively high frequency of adult females and some young females in this habitat supports a possible dominance effect in habitat occupation.

Habitat segregation can also be caused by different dietary requirements or habitat preferences of sex and age classes [11]. We cannot rule out this possibility for young and adult Rusty Blackbirds regarding pecan groves as young were consistently rare in this habitat. However, a reasonably high proportion of adult females were found in pecan groves each year. Given the openness of the habitat and possibly high intraspecific and interspecific competition, it is unlikely that females would use this habitat if it does not suit their requirements. Nonetheless, food resources differed substantially in quality between the three habitats with decreasing abundance of nuts and increasing abundance of invertebrates from pecan groves to forests. This raises the question whether Rusty Blackbirds in different habitats ingest nuts and invertebrates in different proportions. Future studies should address this point.

Habitat quality may not only be determined by food availability but also by predation pressure. We did not measure predation pressure directly but telemetry work conducted at the same time indicated that predation does not seem to be higher in pecan groves than the other habitats (C. Mettke-Hofmann unpubl. data). Therefore, partially harvested pecan groves seem to be high-quality habitats which are primarily used by adult male Rusty Blackbirds. The exclusion of other age and sex classes may be due to intraspecific and/ or interspecific competition.

### Body condition

Body condition has been shown to be a good predictor of survival and habitat quality [45, 46, 47]. In our study, body condition of males was generally higher than that of females, even for individuals captured in the same habitat. This may be another indication of competition between the sexes. Alternatively, baiting may have affected body condition between sexes differently due to better competitive abilities of males. However, from telemetry work we know that individuals only visit the feeding plot occasionally during the day. Furthermore, the two unbaited sites showed the same difference in body condition between sexes as the baited sites making an influence of baiting on body condition unlikely. Young females were in lower body condition than adult females, again presumably due to competition. Alternatively, it may be caused by lower foraging efficiency of young females [48] though this seems to be unlikely as we would otherwise expect the same age-related inefficiency in young males. In contrast, body condition differed only slightly between habitats and was mainly driven by better body condition of males in pecan groves as compared to males in other habitats. It is possible that densities in the different habitats were close to an ideal free distribution [49, 50]. This should be investigated in future studies as we did not measure densities.

Body condition in males correlated with fat and pectoral muscle breadth. While higher fat reserves are often interpreted as an insurance against unpredictable resource availability [51], pectoral muscle breadth accurately reflects flight muscle mass which acts as a source for increased protein demands (e.g. during migration) or during periods of decreased protein availability [33]. Rusty Blackbirds cannot open pecan nuts but are dependent on Common Grackles and other wildlife cracking nuts to get access to the food source which may introduce some unpredictability. Greater fat reserves may lessen the effect of unpredictability and give males an advantage over females. However, the larger pectoral muscle in males clearly indicates better foraging conditions possibly due to competitive abilities. Better body condition in winter has been related to earlier departure to and earlier arrival at the breeding grounds [52], better condition during migration [12] and higher survival to the next year [45] in some songbird species. Therefore, the generally lower body condition of females can have negative carry-over effects into the next breeding season for Rusty Blackbirds.

### Conservation implications

Bottomland hardwood forests are important habitats for a variety of bird species for breeding but also for overwintering. Recent management efforts have primarily targeted breeding Neotropical migrants [53], whereas winter habitat management is primarily directed to water fowl [54]. Zeller and Collazo [55] suggested that conservation efforts should be redirected to resident and temperate migrants in bottomland hardwoods. Rusty Blackbird could be the target of such a conservation effort and an ideal indicator of its effectiveness.

Our food availability analyses show two main results; 1) Nut availability in forests is low as compared to more anthropogenic influenced habitats and 2) hydrology is an important factor as differences in invertebrate biomass between habitats were primarily driven by aquatic and semi-aquatic invertebrates in wet samples. Regarding the first result differences in nut abundance are even larger when we consider density of pecan/ acorn producing trees in the different habitats (61 trees/ha on our pecan grove sites versus 34 trees/ha in natural forests (Nuttall, Water and Willow Oak; [56])). The low nut biomass in forests may be due to selective logging of oak trees in historic times and reforestation that is unlikely to reinstate original structure and composition [57], reforestation with a mixture of oak and fast growing trees [58] and competition with large mammals which are absent from pecan groves and creeks. While reforestation efforts in the Lower Mississippi Alluvial Valley have been dominated by oak species, fast-growing Cottonwood (*Populus deltoides*) and other tree species which provide suitable breeding habitats for several bird species at an earlier time than oaks [53] have also been used [58, 59, 60]. However, a higher percentage of hard-mast producing species (oak and pecan) in forest restoration, particularly small acorn-producing species such as Willow Oak and Water Oak but also native Pecan would provide valuable winter food not only for Rusty Blackbirds but also for water fowl of conservation concern [29, 61], woodpeckers (*Picidae*) and large mammals. In terms of Rusty Blackbird improvement of food availability in forests would specifically improve conditions for females, particularly young ones.

Restoration of forest also requires managing water bodies which affects availability of wet sites. The large differences in the presence of wet sites between the three habitats and as a consequence in invertebrate abundance in wet samples underline this. These wet sites are of particular importance for Rusty Blackbirds as they mainly forage along edges of ponds and streams [20]. Aquatic invertebrates respond with two strategies to retreating water bodies; they either follow the water line or emerge from the soil which exposes them to predators [44]. Similar to water fowl [62], Rusty Blackbirds prefer areas with fluctuating water levels and follow the retreating water edge while foraging (C. Mettke-Hofmann personal observation, [43]). Fluctuating water levels allow these birds to exploit sites more efficiently. Batema *et al*. [43] found that invertebrate abundance is higher in naturally flooded bottomland hardwood sites than in greentree reservoirs which have prolonged periods of flooding and deeper water levels. Flooding regimes with shorter cycles and shallow water would improve feeding conditions in the forest for animals such as the Rusty Blackbird and would also help tree saplings to establish [63].

Riparian forests are important habitats for forested wetland birds [64] but often suffer from edge effects and studies usually recommend having a larger area forested along rivers [64, 65]. Forest fragments along creeks seem to provide a particularly suitable habitat for young male Rusty Blackbirds. However, the combination of relatively high nut as well as invertebrate biomass with the availability of understory made this habitat suitable for all age and sex classes. A broadening of the streamside zone of forest along the creek would definitely improve this habitat further.

Finally, pecan groves seem to be high-quality habitats for adult males and are of great importance as they provide suitable habitat in addition to forests. However, pecan groves are under threat of replacement for bio-fuel production (own obs. C. Mettke-Hofmann). Within our study region several of the pecan groves where we captured birds have already been cleared. To improve conditions of Rusty Blackbirds in pecan groves we recommend sustaining existing groves and/ or instituting programs to support less intensive use of pecan nuts (i.e. part of the harvest should be left on the ground).

## Supporting Information

S1 Table Capture and body condition data. (XLS)

S2 Table Food availability data. (XLS)

S3 Table Legend for S1 and S2 Tables. (DOC)
