# Supplementary material for: Competition and Habitat Quality Influence Age and Sex Distribution in Wintering Rusty Blackbirds
Source: PLoS One. 2015 May 6;10(5):e0123775. doi: 10.1371/journal.pone.0123775 (PMC4422684; doi:10.1371/journal.pone.0123775)
Supplement: S3 Table — (DOC) [file pone.0123775.s003.doc]

**Legends for S1 and S2**

**S1: Capture data**

Location: Capture site

Habitat: 1=pecan; 2=Creek, 3=forest

Year: 1=2005/06; 2=2006/07; 3=2007/08

Day: Capture date

Time: Capture time

Sex: 1=male; 2=female

Age: 1=under 1 year; 2=older than 1 year

Bandno: USGS leg band

Fat_total: average fat of furcula and abdomen

Pectoral_muscle: body condition measured with muscle metre

Tarsus: tarsus length (mm)

Winglength: wing length (mm)

Bodymass: body mass (g)

**S2: Food availability data**

Location: Sampling site

Habitat: 1=pecan; 2=Creek, 3=forest

Year: 4: 2008/09; 5=2009/10

Biomass_inv_mean: mean dry biomass of all invertebrates from dry and wet samples

Biomass_inv_wet: mean dry biomass of all invertebrates from wet samples

Biomass_inv_dry: mean dry biomass of all invertebrates from dry samples

Biomass_nut: mean dry biomass of all nuts
